# Patient Triage by Topic Modeling of Referral Letters: Feasibility Study

**DOI:** 10.2196/21252

**Published:** 2020-11-06

**Authors:** Irena Spasic, Kate Button

**Affiliations:** 1 School of Computer Science & Informatics Cardiff University Cardiff United Kingdom; 2 School of Healthcare Sciences Cardiff University Cardiff United Kingdom

**Keywords:** natural language processing, machine learning, data science, medical informatics, computer-assisted decision making

## Abstract

**Background:**

Musculoskeletal conditions are managed within primary care, but patients can be referred to secondary care if a specialist opinion is required. The ever-increasing demand for health care resources emphasizes the need to streamline care pathways with the ultimate aim of ensuring that patients receive timely and optimal care. Information contained in referral letters underpins the referral decision-making process but is yet to be explored systematically for the purposes of treatment prioritization for musculoskeletal conditions.

**Objective:**

This study aims to explore the feasibility of using natural language processing and machine learning to automate the triage of patients with musculoskeletal conditions by analyzing information from referral letters. Specifically, we aim to determine whether referral letters can be automatically assorted into latent topics that are clinically relevant, that is, considered relevant when prescribing treatments. Here, clinical relevance is assessed by posing 2 research questions. Can latent topics be used to automatically predict treatment? Can clinicians interpret latent topics as cohorts of patients who share common characteristics or experiences such as medical history, demographics, and possible treatments?

**Methods:**

We used latent Dirichlet allocation to model each referral letter as a finite mixture over an underlying set of topics and model each topic as an infinite mixture over an underlying set of topic probabilities. The topic model was evaluated in the context of automating patient triage. Given a set of treatment outcomes, a binary classifier was trained for each outcome using previously extracted topics as the input features of the machine learning algorithm. In addition, a qualitative evaluation was performed to assess the human interpretability of topics.

**Results:**

The prediction accuracy of binary classifiers outperformed the stratified random classifier by a large margin, indicating that topic modeling could be used to predict the treatment, thus effectively supporting patient triage. The qualitative evaluation confirmed the high clinical interpretability of the topic model.

**Conclusions:**

The results established the feasibility of using natural language processing and machine learning to automate triage of patients with knee or hip pain by analyzing information from their referral letters.

## Introduction

### Background

Currently, a pathway recommended for musculoskeletal conditions such as knee or hip pain consists of their management within primary care followed by referral to a multiprofessional assessment and treatment clinic if a specialist opinion is required [1]. The aging population increases the demand for health care resources [2], emphasizing the need to streamline care pathways to maximize efficiency and ensure patients receive optimal care for their needs. With this aim, referral prioritization systems were developed for hip and knee pain and tested to fast-track cases for surgical opinion based on referral information provided by the primary care [3,4]. However, their prioritization criteria lacked adequate sensitivity and specificity for patients moving between surgical and conservative pathways. Information conveyed in referral letters underpins the referral decision-making process, but it has not been explored systematically for the purposes of treatment prioritization for musculoskeletal conditions. Automated analysis of referral letters can identify variables that can be used alongside demographic and health-related data to improve treatment prioritization. Within the context of musculoskeletal conditions, natural language processing (NLP) was used successfully to automate the analysis of radiology reports [5,6] and patient questionnaires [7].

Indeed, NLP has repeatedly demonstrated its feasibility to extract clinical variables from clinical narratives, making them available for large-scale analysis down the stream [8]. Traditionally, rule-based approaches have been commonly used to extract variables of predefined types [9]. Machine learning has long been hailed as a silver bullet solution for the knowledge elicitation bottleneck, the main argument being that the task of annotating the data manually is easier than that of eliciting the knowledge. However, a recent systematic review of machine learning approaches based on clinical text data revealed the data annotation bottleneck to be one of the key obstacles to machine learning approaches in clinical NLP [10]. However, the biggest challenge for these applications to become part of routine clinical practice is the problem of human interpretability of automated outputs. Machine learning approaches may offer faster development of algorithms and their performance improvement, but some do so at the expense of the interpretability of the results [11]. Topic modeling can kill both birds with one stone. First, the aim of topic modeling is to identify latent topics that can be used to organize a corpus, where each document contains a mixture of topics in different proportions. As an unsupervised method, it does not require data to be annotated manually. This means that the algorithm can readily utilize vast amounts of data, allowing the machine learning model to more accurately capture statistically significant patterns. Second, each topic is associated with a set of words that are extracted automatically from the corpus based on their distribution. The highest-ranked words can help interpret the underlying semantics.

### Related Work

A popular topic modeling algorithm is the latent Dirichlet allocation (LDA) [12]. LDA is a three-level hierarchical Bayesian model in which each document is modeled as a finite mixture over an underlying set of topics and each topic is modeled as an infinite mixture over an underlying set of topic probabilities. Although LDA is used frequently in NLP research, it is yet to make a significant mark on clinical NLP, which is still heavily biased in favor of supervised learning methods [10]. Nonetheless, LDA is steadily finding its clinical applications, such as improving clinical process efficiency [13-15], predicting hospital readmission [16], patient safety [17-19], and patient phenotyping [20-22]. Some of the topic models were specifically evaluated for interpretability from a clinician's perspective [14,16]. To improve coherence and interpretability of topics, some approaches combined LDA with clinical terminologies, such as the Medical Dictionary for Regulatory Activities [18] and the Systematized Nomenclature of Medicine Clinical Terms [15]. Typical reasons cited for choosing LDA over supervised learning approaches include alleviating the need for labor-intensive data annotation, avoiding human annotation bias, and the potential to identify latent topics in the data that may not be apparent a priori. The latter is particularly important in clinical scenarios with *unknown unknowns*, such as patient safety [17-19]. In terms of training a topic model, many approaches struggled to fine-tune the number of topics as one of the key hyperparameters of the LDA algorithm. In most cases, a plausible justification for the number of topics was lacking, for example, 25 [20], 100 [17,18], 75 [16], 50/100/150 [14], and 50/100/200 [21].

The research gaps identified in this overview of related work are as follows. Despite finding various clinical applications, LDA is yet to be used to support triage. The biggest challenge for these applications to become widely adopted in clinical practice is the perception of interpretability. However, few studies have specifically evaluated the interpretability of the LDA outputs from a clinician's perspective. Clinical terminologies have been combined with the LDA to improve interpretability, but the resources used to support such functionality do not include the Unified Medical Language System (UMLS), which offers a unique opportunity to abstract clinical concepts into higher categories of knowledge. Finally, for the topics to be easily distinguishable (and, hence, interpretable), their number needs to reflect the latent themes and patterns present in a given data set. However, none of the considered approaches provided a strategy to infer the value of this hyperparameter from the data. In this study, we addressed these four gaps.

First, we applied the LDA to a corpus of referral letters and used topics as features to automatically classify each letter against a list of potential treatments. This can then be used to automate patient triage, that is, assort them into priority groups according to their medical needs. Second, we proposed a novel method for evaluating the interpretability of topics. Third, we used the UMLS to incorporate the interpretation of clinical concepts at different levels of abstraction into the LDA. Finally, we systematically fine-tuned the number of topics using a measure of topic coherence.

## Methods

### Data Collection

Data collection was originally described in the study by Button et al [23]. In summary, patients were eligible to take part in the study if they were referred by their general practitioner for joint (knee or hip) pain, they were aged 18 years or older, they could provide informed consent, and they could speak English fluently. The exclusion criteria included pain secondary to other health conditions such as rheumatoid arthritis, pain secondary to joint replacement, surgery for the same joint within the last 12 months, or having already received treatment at the primary-secondary care interface for the same condition within the last 6 months.

**Figure 1:**
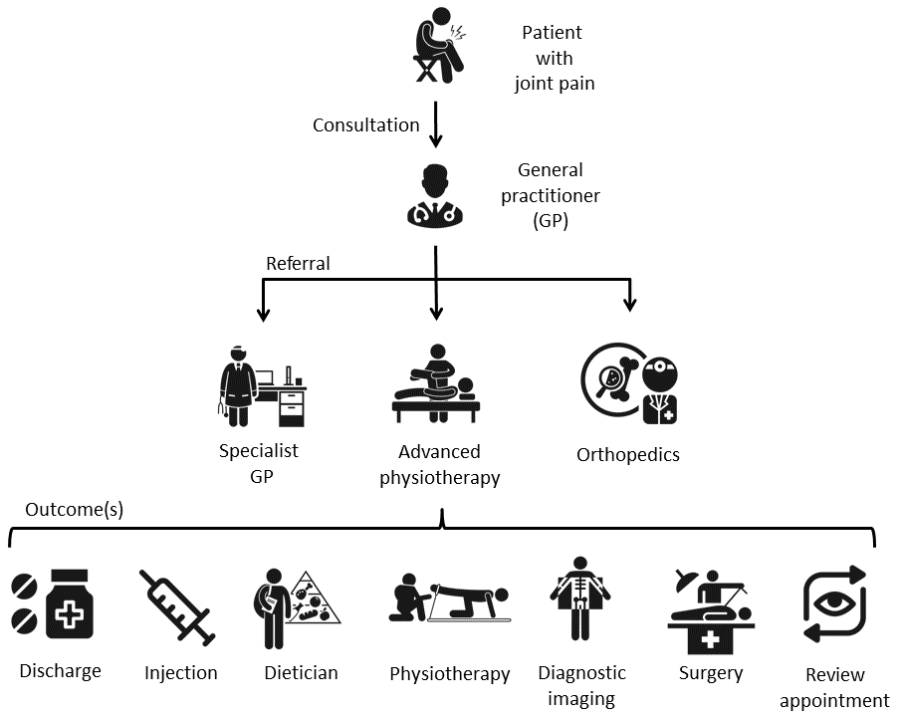
Musculoskeletal care pathway for adults with hip and knee pain. GP: general practitioner.

The care pathway is illustrated in Figure 1. A patient with joint pain is referred by a clinician from their general practice to a specialist clinic in secondary care, which could be an orthopedic clinic, general practice with musculoskeletal specialism, or advanced physiotherapy clinic. Appropriate treatment is suggested when the patient is seen in secondary care.

Patients were recruited from one Local Health Board, an administrative unit within the National Health Service in Wales, which supports a population of around 445,000 people. A total of 634 participants were recruited between August 2016 and January 2017, and their referral letters were collected. The follow-up data collection was completed in June 2018. This included recording of any treatments performed. A subset of 576 patients with complete data, including the original referral letter and the corresponding treatments, was used in this study. The distribution of their treatments is given in Table 1. Note that a single patient may have had multiple treatments.

**Table 1:** The distribution of treatment referrals.

ID	Treatment	Total number of patients, n
O1	Orthopedic referral	53
O2	Discharge (no further appointments booked)	173
O3	Injection	101
O4	Nutritionist	15
O5	Physiotherapy	152
O6	Diagnostic imaging	112
O7	Surgery	99
O8	Review appointment	223
O9	Any other referral	16

### System Design

**Figure 2:**
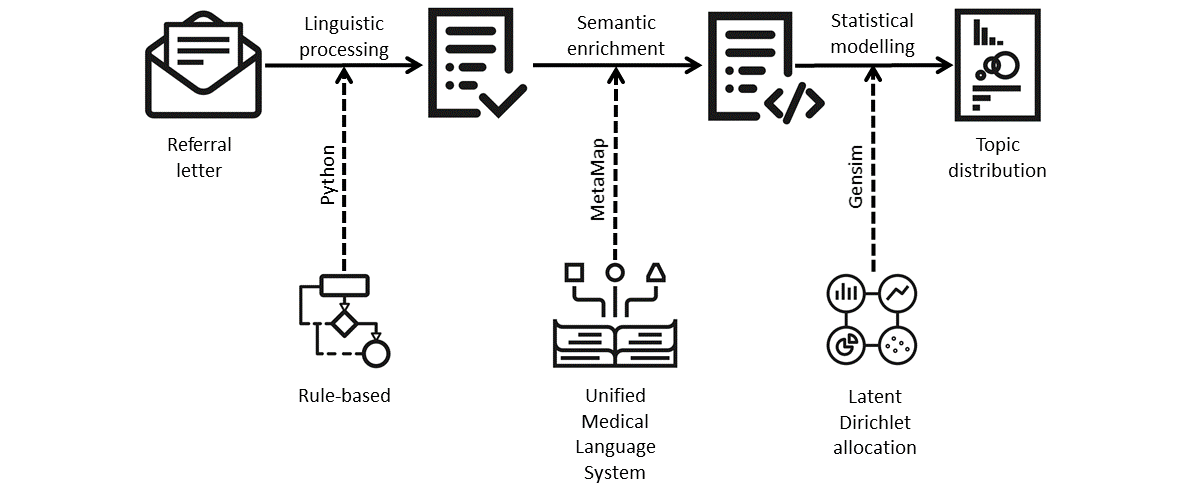
System design for topic modeling of referral letters.

The main research question addressed in this study is as follows: Can triaging patients (into cohorts) based on their referral letters be semiautomated? To that end, we designed a system that can support referral decision making (Figure 2). A corpus of referral letters was used to train a topic model with the ultimate aim of using topics to narrow down the choice of potential treatments and streamline the referral pathway. To reduce potential overfitting to a relatively small training data set, we regularized and generalized its text content. First, the text was regularized by applying a set of linguistic rules designed to reduce idiosyncrasies associated with clinical sublanguage, covering punctuation, acronyms, abbreviations, orthographic and lexical variation, and personal names of patients and clinicians. Subsequently, an external medical language system was used to effectively normalize the terminology used, making the topic model robust with respect to terminological variation. The following sections describe the three modules in greater detail.

### Linguistic Processing

The linguistic preprocessing and normalization module originally developed to support cohort selection from hospital discharge summaries was adapted for this study [24]. In addition to standard linguistic preprocessing operations, this module also handles punctuation in clinical narratives, which can affect the results of text segmentation algorithms developed for general language [25]. However, its main purpose is to streamline subsequent text analysis and reduce overfitting by regularizing the text content. This involves basic string operations such as lowercasing, fully expanding enclitics, and special characters. It further normalizes text content by replacing a selected subset of words and phrases with their representatives. Here, special consideration is given to acronyms and abbreviations as they are known to have a major impact on the retrieval of relevant information [26]. These mappings are supported by a set of local lexica whose content was adapted for this study to support migration from the domain of hospital discharge summaries to that of referral letters. To facilitate this process, we extracted multiword terms (including their acronyms) from referral letters automatically using FlexiTerm [27,28] and manually curated the list of conflated term variants.

New functionality added to the linguistic processing module includes recognition of personal names. Personal names, like any other words, can be selected automatically as topic descriptors. For example, if several patients were referred to Dr Jane Doe, who is a physiotherapist, then her name may become correlated with a *physiotherapy theme* in referral letters, ultimately resulting in the words “Jane” and “Doe” emerging as the topic descriptors. Not only are these words not informative of the topic but they also cannot be generalized to other data sets where these names do not exist, or they refer to different persons, thus rendering the model either inapplicable or inaccurate. To prevent a topic model from overfitting to personal names, they are replaced by a generic representative. For this purpose, we originally considered existing named entity recognition libraries (eg, [29,30]) to recognize personal names in referral letters. However, having been designed with general language in mind, their overzealous matching algorithm could not distinguish between different uses of personal names. As illustrated by the taxonomy for the rehabilitation of knee conditions [31], many clinically relevant concepts feature personal names, for example, Hoffa fat pad, Baker cyst, or McMurray test. Replacing these mentions of personal names with generic representatives would remove important content that can be used to describe a topic. On the other hand, referral letters are written using a formal style, which prescribes the use of honorifics. This fact was exploited to define a set of regular expressions based on honorifics and capitalization of personal names to automatically recognize the names of patients and clinicians. These names were replaced with a generic representative. This approach preserved personal names used to name body parts, diseases, tests, and any other medical concepts.

### Semantic Enrichment

As a statistical model, a topic model may benefit from aggregating the distribution of synonyms (eg, “physio” and “physiotherapy”). Linking synonyms gives the model a better chance of capturing the semantics of underlying topics. Linguistic preprocessing implements lexical normalization, where both formal and informal abbreviations are translated to a standard vocabulary. For instance, “TKR” and “physio” would be translated to “total knee replacement” and “physiotherapy,” respectively. However, the problem of term variation may still persist. Examples from our corpus are many: “tear” versus “rupture,” “painkiller” versus “analgesic,” “oedema” versus “swelling,” “patella” versus “kneecap,” etc. The UMLS [32], which integrates multiple terminologies, classifications, and coding standards, maps such terms to concepts, which are assigned a concept unique identifier (CUI). A CUI can be used to markup synonymous terms in the text. Consider, for example, the sentences given in Textbox 1. Concept markups can be processed by topic modeling software similar to any other tokens in the corpus and, therefore, can be used as potential topic descriptors.

**Concept markups.**

1. She struggles to take any *painkillers/C0002771* stronger than paracetamol.
2. He is opposed to regular *analgesics/C0002771*.
3. His recent magnetic resonance imaging shows *oedema/C0013604* and bursitis.
4. There is a little bit of *swelling/C0013604* of the knee joint.
5. The magnetic resonance imaging showed a complex *tear/C3203359* of the medial meniscus.
6. She has had a likely anterior cruciate ligament *rupture/C3203359*.

Moreover, concept markup can be used to effectively group together multiword expressions. This may improve the interpretability of topics. For example, when words describing a topic are presented independently of one another, such as “medial,” “joint,” “line,” and “tenderness” instead of “medial joint line tenderness,” then it is unclear whether the word “medial” refers to “meniscus” (“medial meniscus”), “ligament” (“medial collateral ligament”), “condyle” (“medial femoral condyle”) or indeed a “joint line” (“medial joint line”). Similarly, it remains unclear which anatomical entity is affected by “tenderness.” To alleviate this problem, topic modeling approaches often use an *n*-gram language model [33], with *n* being fixed to 2 and 3. Examples from our corpus (Textbox 2) illustrate that an *n*-gram approach may be too rigid for biomedical sublanguage, which is known for its terminological variability [27,28].

**Markup of multiword terms.**

1. I could not reproduce pain with *McMurray test/C3669149*.
2. She does however experience pain on *McMurray* and Ege *testing/C3669149*.
3. He would be keen to consider a *total knee replacement/C0086511* as his pain has increased.
4. She is relatively young for consideration of *knee arthroplasty/C0086511*.
5. She has poor mobility following a few revisions of a right *knee prosthesis/C0086511*.
6. He is a 67-year-old male who has had *bilateral knee pain/C2220048* for a number of years.
7. She has persistent *pain in both knees/C2220048* with regular effusions.
8. She has crepitus in his left knee with *medial joint line tenderness/C0576135*.
9. No swelling of the knee but *tender medial joint line/C0576135*.
10. He had an effusion present and was *tender across his medial joint line/C0576135*.
11. On examination there was *tenderness along the joint line medially/C0576135*.

MetaMap, a highly configurable dictionary lookup software, can be used to discover the UMLS concepts in the text [34]. We used MetaMap to markup concepts such as those presented in Textboxes 1 and 2. Table 2 provides the most relevant details of the MetaMap configuration used. MetaMap also maps concepts to semantic types. Like CUIs, they can be used for markup. Semantic type markups can be used to unify concepts depicting a common theme. As examples from our corpus illustrate (Textbox 3), references to sports activities are very diverse. Individually, they may not be selected as topic descriptors because their occurrences are relatively rare. However, when they are mapped to their semantic type (*daily* *or* *recreational activity* (DORA)), we can observe common themes emerging focusing on age, fitness, and injury: young, physically active patients with a sports-related injury. These factors play an important role in recommending the most appropriate treatments. Their association with the given semantic type means that it could be a useful topic descriptor. For example, a clinician can reasonably assume that the given topic refers to a cohort of young, fit patients with a sports-related injury. Semantic type markups can be processed by topic modeling software similar to any other tokens in the corpus and, therefore, can be used as potential topic descriptors.

**Table 2:** MetaMap configuration.

Parameter	Description	Used	Rationale
a	Allows matching of acronyms and abbreviations.	No	These are the least reliable form of variation, for example, “OA” has got at least three full forms, for example, “osteoarthritis,” “optic atrophy,” and “ocular albinism.” Local lexica were used in linguistic processing module instead to enforce tighter control of acronyms and abbreviations.
i	Ignores word order when matching a text phrase to a candidate concept name.	Yes	This option allows for syntactic variants such as “meniscus tear” and “tear of meniscus” to be conflated.
D	Forces the use of all derivational variants instead of only those between adjectives and nouns.	Yes	This option adds flexibility to conflation of syntactic variants such as torn/VBN meniscus/NN and meniscal/JJ tear/NN.
l	Enables retrieval of candidates for two-character words occurring in more than 2000 UMLS^a^ strings and one-character words occurring in more than 1000 UMLS strings.	No	Like acronyms and abbreviations, short words are highly ambiguous.
8	Generates variants dynamically rather than by a table look up.	Yes	This option adds further flexibility to conflation of syntactic variants.
y	Attempts to disambiguate among concepts scoring equally well in matching input text by choosing concepts having the most likely semantic type in the given context.	Yes	This option supports correct interpretation of certain words, for example, “fall” used in “his pain started in April when he had a fall on his left knee” should be interpreted as “a sudden movement downward, usually resulting in injury” rather than “the season between the autumnal equinox and the winter solstice.”
Y	Favors mappings with more concepts over those with fewer concepts.	No	Instead of fixed *n*-grams, we prefer to identify the longest collocationally stable word sequences, for example, a single concept “ligament tear” instead of 2 separate concepts “ligament” and “tear.” In addition, longer matches also reduce ambiguity, for example, recognizing “tear” as part of “ligament tear” prevents its incorrect interpretation as “the fluid secreted by the lacrimal glands.”
J	Restricts to semantic types in the comma-separated list.	Yes	To reduce the number of incorrect interpretations, we limited concept mappings to a fixed list of most relevant semantic types, which have been selected manually by a clinical expert.^b^

^a^UMLS: Unified Medical Language System.

^b^The full list of semantic types and their mappings is available from MetaMap Documentation [35].

**Markup of semantic types. DORA: daily or recreational activity.**

1. This 22 year old was tackled in *rugby/DORA* [35] and sustained an injury.
2. She is a delightful 27 year old female who when *skiing/DORA* last year felt something pop in her knee.
3. He is normally quite active and enjoys *football/DORA*, which he is now unable to do.
4. It first started about an hour after playing *badminton/DORA*, which is something that he does.
5. He was previously very active and was involved in *sport/DORA* but has been unable to recently.
6. He is a keen *ice hockey/DORA* player.
7. Thank you for seeing this man who two years ago injured his right knee playing *basketball/DORA*.
8. She is a very athletic female, and back in 2013 had a *netball/DORA* injury.
9. It was not caused by trauma, but playing *golf/DORA* worsens it.
10. Patient is normally very fit and active playing *tennis/DORA* on a weekly basis.

### Topic Modeling

To implement our topic modeling approach, we used the LDA method, which discovers latent topics in a corpus of documents based on a Bayesian statistical modeling approach [12]. This approach was chosen to support patient triage for the following reasons. By not fixing patient cohorts in advance, we wanted to avoid the need for manual annotation of data. More importantly, an unsupervised approach can identify previously unobserved patient groups beyond the boundaries of a predetermined classification scheme. Unlike cluster analysis, which can be used to support the same goal, topic modeling allows cluster overlap. This makes the problem of referring patients to multiple treatments easier to model. Interpretation of such a model is supported by (1) word distributions per topic and (2) topic distributions per document.

We used an open-source implementation of the LDA algorithm included in the Gensim library [36]. Each document was represented by a bag of words (BOW), which means that word positions and their local contexts were not taken into account. This can be partly remedied by introducing *n*-grams into the BOW representation. As described earlier, we opted to use tokens that represent markups of concepts and semantic types as an alternative to *n*-grams with added benefits of normalizing lexical and syntactic variation associated with biomedical terms. We ran experiments with different combinations of features, as described in Table 3.

**Table 3:** Data sets used in experiments with different types of features included.

Data set	Words	Concepts	Semantic types
D1	Yes	No	No
D2	Yes	Yes	No
D3	Yes	No	Yes
D4	Yes	Yes	Yes

### Hyperparameter Tuning

The performance of machine learning models depends not only on the parameters whose values the model learns during the training phase (eg, the weights for each word in a given topic) but also on the values of hyperparameters (eg, the number of topics), which are fixed before the training begins. The predictive performance of different topic modeling algorithms was found to vary substantially in practice. However, when the hyperparameters were optimized, these differences diminished significantly [37]. One of the key hyperparameters of the LDA algorithm is the number of topics. The difficulty arises when the number of relevant topics is not known a priori. An insufficient or excessive number of topics could render an LDA model too coarse or overly complex, respectively.

**Figure 3:**
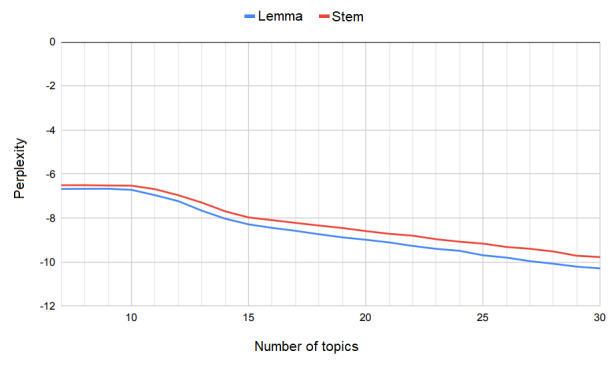
Perplexity as a function of the number of topics.

Perplexity, a measure of how well a probabilistic model predicts a sample, is commonly used to evaluate topic models. It is calculated as the inverse of the geometric mean per-word likelihood, with lower values indicating better models [38]. A heuristic approach based on the rate of perplexity change as a function of the number of topics has been proposed to determine an appropriate number of topics [39]. This approach would suggest selecting 11 as the total number of topics based on the values shown in Figure 3.

**Figure 4:**
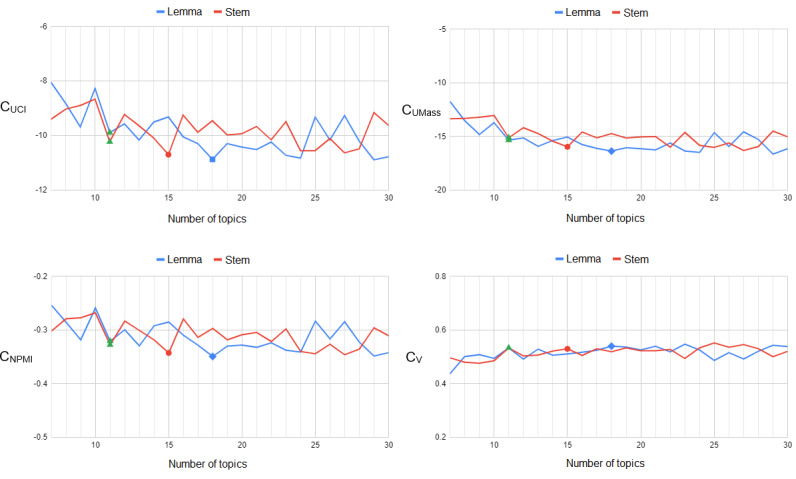
Topic coherence as a function of the number of topics.

In general, perplexity was found not to be well correlated with the human rating of topic interpretability [40]. Alternative measures based on word coherence have been proposed to remedy this problem [41]. We used 4 measures of topic coherence, which are described in more detail in the *Results* section. As Figure 4 illustrates, the coherence of stemmed and lemmatized text achieved an optimum using 15 and 18 topics labeled by red circles and blue squares, respectively. However, at both points, topic coherence demonstrated opposite trends. However, at another local optimum labeled by green triangles, topics modeled on stemmed and lemmatized text demonstrated not only similar trends but also almost identical coherence values. Given a small difference from the global optimum, we selected 11 as the total number of topics to be able to switch freely between stemming and lemmatization in subsequent experiments. This choice also complied with the one based on perplexity.

## Results

### Intrinsic Evaluation

**Figure 5:**
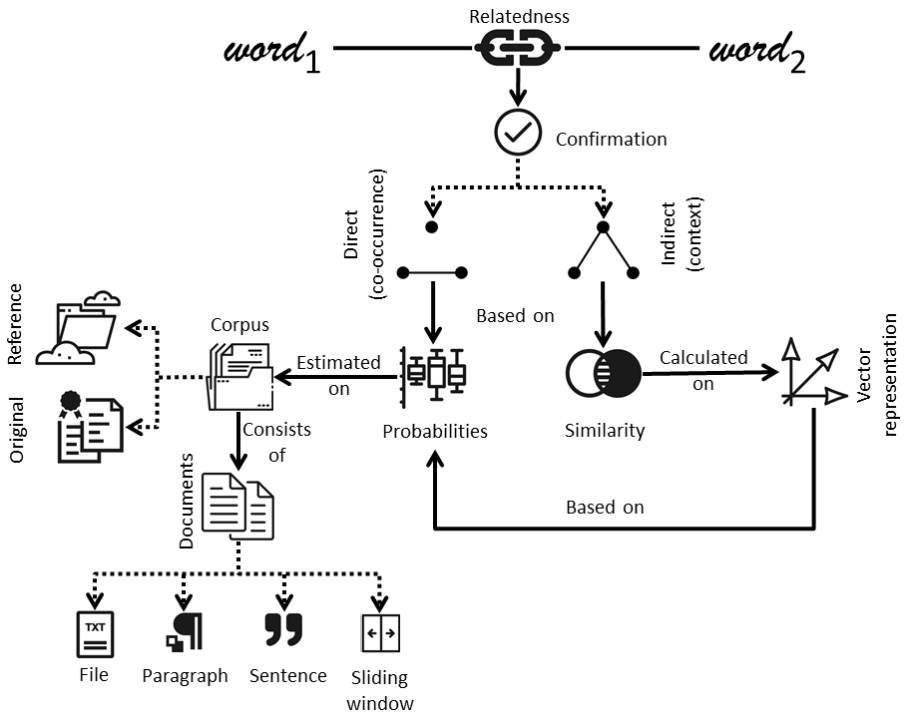
Corpus-based approaches to measuring word coherence.

Recent studies have shown that optimizing a model for perplexity may not yield human interpretable topics [40]. This limitation has prompted further research into alternative ways of estimating human interpretability. Newman et al [42] introduced the notion of topic coherence, which is based on the coherence of words that describe a topic. Different variants of this measure have been proposed [41]. In principle, overall coherence is averaged across word pairs in a topic and then across topics. Therefore, the overall topic coherence depends on the way the coherence between 2 words is measured. Figure 5 focuses on this problem. In principle, coherence refers to the degree to which 2 words are related. Two approaches to measuring relatedness can be used: one based on direct co-occurrence (or collocation) and the other based on co-occurrence with a shared set of other words.

In the first approach, 2 words are said to be collocated if they co-occur more often than would be expected by chance. In corpus linguistics, collocation is measured by estimating relevant probabilities from a corpus of text documents, which can be either the original corpus used to learn the topic model or a reference corpus such as Wikipedia. Probabilities are estimated using Boolean documents. The number of documents in which the word (or a pair of words) occurs is divided by the total number of documents. Neither the number of occurrences within a document nor the distances between words are taken into account; hence, the name Boolean. A virtual document can be defined as a paragraph, sentence, or text window, which, by being smaller parts of the whole document, indirectly account for the distances between words.

These probabilities are used to calculate pair-wise word coherence measures such as pointwise mutual information (PMI) [43], normalized pointwise mutual information (NPMI) [44], or log-conditional probability (LCP) [45] as follows (small positive is added to avoid logarithm of zero):


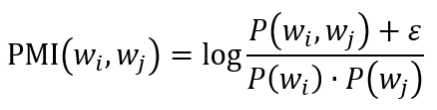



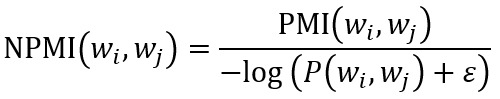



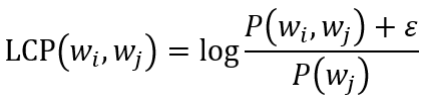


PMI compares the probability of 2 words co-occurring, *P*(*w_i_, w_j_*), against the probability that they would co-occur under the assumption of their independence, *P*(*wi*)*P*(*w_j_*). Higher values indicate a stronger association between the 2 words. NPMI follows the same logic, but it also imposes a fixed upper bound of 1 to indicate perfect association by normalizing PMI using the joint probability of 2 words. This makes its interpretation more intuitive while also reducing the bias toward less frequently occurring words. Both measures are symmetric, which is not a property of human word associations. By basing LCP on a simple conditional probability *P*(*w_i_* | *w_j_*), it adds direction to measuring the association of 2 words.

Topic coherence is calculated by averaging the pair-wise word coherence across its *n* words:


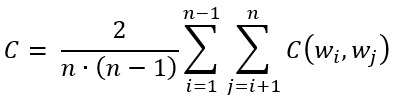


Topic coherence measures based on PMI, NPMI*,* and LCP are commonly referred to as *C*_UCI_ (or *C*_PMI_) [42], *C*_NPMI_ [46], and *C*_UMass_ [47], respectively. The problem with these measures is that they may fail to identify synonyms as related words as they do not co-occur regularly. However, we can reuse any of the pair-wise word coherence measures to represent each word *w_i_* as a vector whose *j*-th coordinate corresponds to *C*(*w_i_*, *w_j_*). On the basis of the distributional hypothesis, which states that words with similar distributions have similar meanings, we can use cosine similarity between the corresponding vectors to estimate the similarity between 2 words:


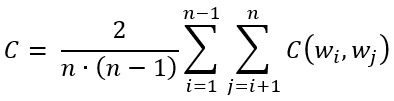


Topic coherence can now be calculated by averaging the contextual similarity across its *n* words [46]:


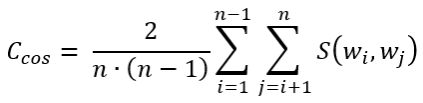


In a comparative analysis, the best correlation with human topic coherence ratings was achieved with *C*_V_ [41], a topic coherence measure that uses cosine similarity on context vectors based on *C*_NPMI_ but differs from *C*_cos_ in a way in which it aggregates the similarity values. Instead of pair-wise comparison, each word is compared with the set of top-ranked words whose context vectors have been summed up.

The Gensim library [36], which was used to create topic models, was also used to calculate their coherence. It implements 4 coherence measures: *C*_UCI_ [42], *C*_NPMI_ [46], *C*_UMass_ [47], and *C*_V_ [41]. Table 4 reports their values obtained for topic models extracted from the data sets described in Table 3. Overall, the best results were achieved on data set D2, which was obtained by annotating the original text with concepts from the UMLS.

**Table 4:** Topic coherence.

Data set	*C*_UCI_	*C*_NPMI_	*C*_UMass_	*C*_V_
D1	−9.89	−0.32	−15.34	0.53
D2	−12.23	−0.41	−17.31	0.68
D3	−10.68	−0.35	−17.50	0.59
D4	−11.12	−0.37	−17.12	0.59

### Extrinsic Evaluation

**Figure 6:**
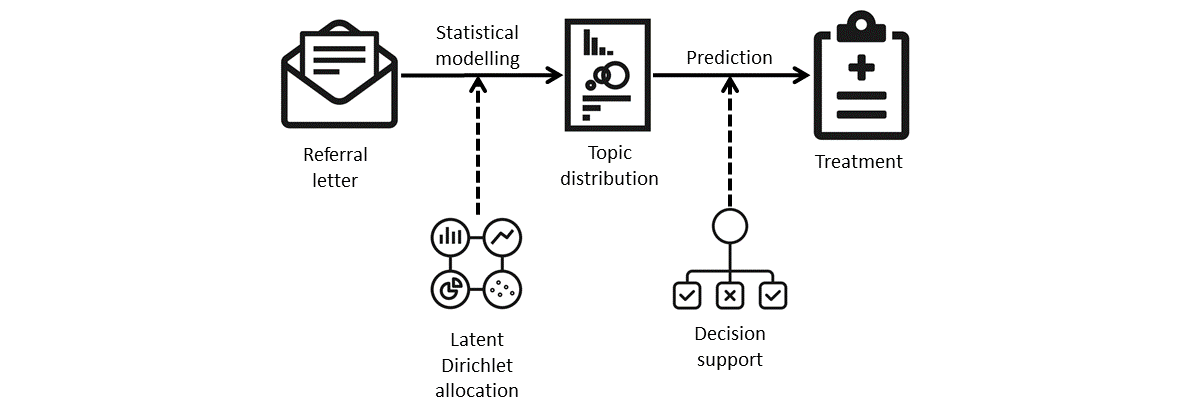
Supporting patient triage with topic modeling.

The extrinsic evaluation assesses the performance of a topic model in the context of a predefined task. In an envisaged scenario, topic modeling could be used to semiautomate patient triage by using topics to predict the most appropriate treatments (Figure 6). Our data set included the referral letters together with subsequently received treatments (Table 1).

As a result of topic modeling, each referral letter was mapped to a topic distribution vector. Each coordinate contained a score that the letter received against the corresponding topic. Effectively, the corpus was transformed into a document-topic matrix. We trained a binary classifier for each treatment using the document-topic matrix. It takes a topic distribution vector of a referral letter as input and outputs a yes or no decision for the corresponding treatment.

**Figure 7:**
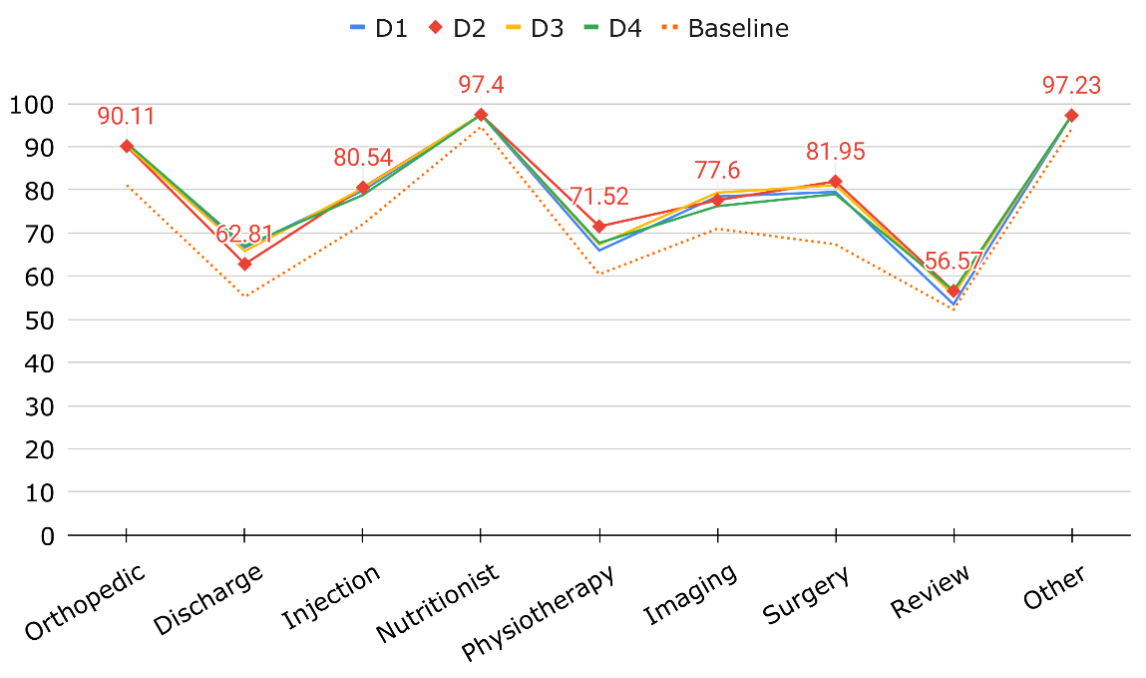
Predictive accuracy of a classifier trained on top of a topic model.

We used 10-fold cross-validation to measure its prediction accuracy A=(TP+TN)/N, which was calculated using true positives (TP), true negatives (TN), and the total number (N). Cross-validation experiments were performed for each data set described in Table 3. Given a small number of features combined with few instances of some treatment outcomes, we opted for the *k*-nearest neighbor algorithm with *k*=5 in a quest to reduce overfitting. The cross-validation results are shown in Figure 7.

Not surprisingly, the worst results were achieved on discharge and review appointment. One would intuitively expect that these outcomes would be the least homogeneous with respect to topic distribution. In other words, any musculoskeletal patient would eventually be either discharged or reviewed, regardless of their condition. The best results were achieved for the 2 most imbalanced treatment outcomes, Nutritionist and Any other referral, with only 15 and 16 positive instances, respectively, out of a total of 576, where overfitting the majority class was most likely to have occurred. The accuracy of predicting the remaining treatment outcomes outperformed the stratified random classifier by a large margin, indicating that topic modeling could be used to support patient triage (Figure 6). On average, the best accuracy was achieved on data set D2, which augments the raw text features with domain-specific concepts. The best performance is in line with the best topic coherence recorded in the intrinsic evaluation (Table 4).

### Qualitative Evaluation

**Figure 8:**
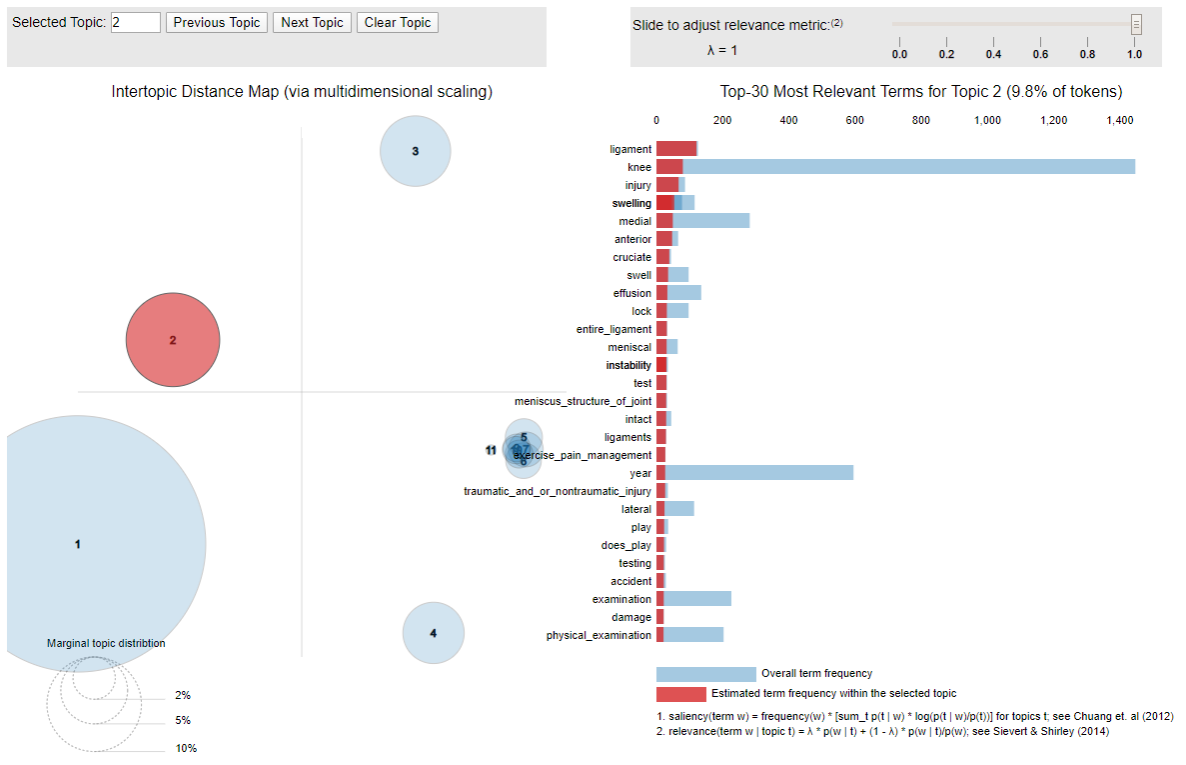
Interactive visualization of a topic model.

Qualitative evaluation is de facto the gold standard for measuring the interpretability of a topic model. However, involving human raters makes such an evaluation expensive to implement in practice. For that reason, we singled out a topic model with the highest coherence (Table 4) and classification accuracy (Figure 7) for further evaluation with respect to its interpretability. Its interactive web-based visualization (see Figure 8 for an example) was created using pyLDAvis, a Python library designed to help users interpret a set of latent topics [48]. Each topic was represented by a circle whose size reflects its prevalence in the training corpus. The distance between the centers of the 2 circles reflected the similarity between the corresponding topics. Clicking on a circle resulted in a histogram of the top 30 words most relevant to the corresponding topic. Here, relevance was determined based on a parameter (0 1). By default, λ was set to 1 to rank the words by their probability within a topic. When λ was set to 0, the words were reranked by their lift, which is defined as the ratio of a word's probability within a topic to its marginal probability across the corpus. The interactive interface allowed a user to adjust the value of λ between 0 and 1.

**Figure 9:**
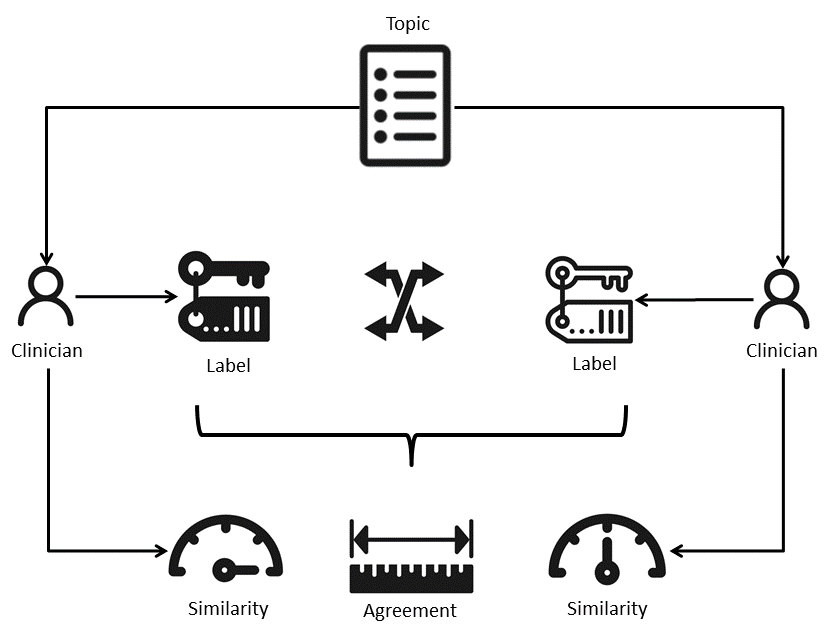
Experimental protocol for measuring topic interpretability.

To measure the interpretability of topics, we designed experiments using a novel protocol illustrated in Figure 9. In this scenario, 2 medical doctors with specialization in physiatry were paired. Independently, each clinician was presented with an interactive visualization of the topic model (Figure 8). They completed a survey in which they were asked to describe each topic using a short free-text statement that generalizes the collective meaning of the topic's 30 most relevant words as a cohort of patients. No restrictions were imposed on the facets used in their description (eg, age, fitness, or pathology) or the choice of vocabulary. Although describing individual topics, the 2 clinicians were also asked to estimate the confidence in their final choice on a 5-point Likert scale: 0 (not confident at all), 1 (slightly confident), 2 (somewhat confident), 3 (moderately confident), and 4 (very confident).

In the second phase, both clinicians gained access to the other one's choice of a topic's description. They were then asked to independently estimate the similarity of the 2 descriptions on a 6-point Likert scale: −3 (very dissimilar), −2 (moderately dissimilar), −1 (slightly dissimilar), 1 (slightly similar), 2 (moderately similar), and 3 (very similar). The average similarity was used to estimate the interpretability of topics under the hypothesis that high similarity implies high interpretability and vice versa. The responses to the 2 questionnaires are presented in Table 5.

**Table 5:** The responses to topic interpretability questionnaires.

Topic and description		Confidence	Similarity
**T1**			
	Symptomatic degenerative conditions related to the musculoskeletal system, most commonly the knee and predominantly in females.	Moderately confident	Moderately similar
	Chronic knee pain caused by an injury, causing problems for months and with a positive medical history. Related to women, medial side, and examined by x-ray. In addition to injury, chronic diseases include osteoarthritis, which can be examined by radiological diagnosis and physical examination, which reduces the range of motion and the ability to walk, and which can be treated with physical therapy and other procedures to reduce the feeling of pain.	Moderately confident	Very similar
**T2**			
	Knee ligament injuries with a description of the type of ligament and associated symptoms, most commonly effusion.	Moderately confident	Very similar
	Traumatic and nontraumatic injuries of knee ligaments, especially the medial and anterior cruciate ligaments, with swelling, effusion, and the involvement of the entire ligament leading to instability and locking of the knee. The entire ligamentous apparatus and menisci need to be tested. A history of recurrent injuries plays a role in the damage. Exercise and pain management are recommended.	Moderately confident	Very similar
**T3**			
	Diagnosis of the pathological condition predominantly by magnetic resonance imaging together with a description of the knee injury type.	Moderately confident	Very similar
	Magnetic resonance imaging used to diagnose mostly knee damage, thinning of cartilage, lateral ligaments, and hyaline and less for facets, fissures, and patellar problems.	Moderately confident	Very similar
**T4**			
	Pathological conditions related to the hip.	Moderately confident	Moderately similar
	Degenerative changes of the hip diagnosed by x-ray imaging, hip pain, decreased mobility, and reduced joint space, possibly requiring a hip replacement. Osteoarthritis diagnosed from jagged edges and anti-inflammatory processes. All these changes lead to a decreased range of motion and depression.	Somewhat confident	Very similar
**T5**			
	Coping with sports injuries related to the musculoskeletal system.	Moderately confident	Very similar
	Sports injury mostly caused by twisting. Treated with ibuprofen and bracing. Diagnosed by radiography.	Moderately confident	Very similar
**T6**			
	Medications for painful conditions of the musculoskeletal system.	Moderately confident	Very similar
	Knee injuries treated with a variety of medications.	Somewhat confident	Very similar
**T7**			
	Musculoskeletal condition (knee) that requires an invasive procedure.	Moderately confident	Very dissimilar
	Injuries that occur due to obesity and inactivity.	Slightly confident	Moderately dissimilar
**T8**			
	Degenerative changes in the musculoskeletal system resulting in reduced activity and comorbidities.	Moderately confident	Moderately similar
	Cardiovascular diseases associated with chronic lung disease, hypertension, coagulation disorder.	Somewhat confident	Slightly similar
**T9**			
	Musculoskeletal condition (knee) more often in the female population.	Somewhat confident	Moderately similar
	Most commonly, popliteal cyst, a predisposition in occupations that require prolonged standing, can lead to knee deformities. Excision is a recommended treatment.	Slightly confident	Very similar
**T10**			
	Pain in the lumbosacral spine.	Somewhat confident	Very similar
	Changes in the lumbar spine and pelvis due to osteoarthritis and infection. Accompanied by hot, burning back pain and progression.	Slightly confident	Very similar
**T11**			
	Patients with amputation of the lower extremities.	Moderately confident	Very dissimilar
	Poor mobility due to asymmetries.	Slightly confident	Very dissimilar

The average confidence was found to be 3.00 and 2.00 between the two annotators. The average similarity was found to be 2.00 for both annotators. One participant was consistently more confident than the other, but they were mostly not more than one Likert point apart. The biggest discrepancy between the 2 Likert points was found for topics T8 and T11. When cross-referenced against the topic similarity scores, most dissimilar descriptions were observed. Overall, the participants' perception of topic similarity was consistent, with one Likert point difference throughout.

To generalize these findings, we calculated the interannotator agreement for both confidence and similarity (Table 6). For this purpose, we used Cohen kappa coefficient with linear weighting [49-52]. The agreement on confidence was low. However, a closer look at the distribution of confidence scores between the 2 participants revealed that one participant was consistently more confident than the other. Therefore, the low agreement on confidence in interpreting the topics was more likely to be associated with the participants' own characteristics than the topics themselves. Indeed, the participant with higher confidence provided more generic descriptions, whereas the other paid more attention to detail, which may have lowered their confidence in believing that they addressed the task effectively. Nonetheless, in the vast majority of cases (9 out of 11 topics), the high similarity scores indicate that both generic and detailed descriptions effectively referred to the same cohort, that is, a group of patients who share common characteristics or experiences such as medical history, demographics, and possible treatments. Therefore, based on the hypothesis that high similarity implies high interpretability and vice versa, we conclude that the given topic model was highly interpretable.

**Table 6:** Interannotator agreement on topic description.

Characteristics	Confidence	Similarity
Observed kappa	0.1391	0.7343
Standard error	0.0925	0.1163
Confidence interval	0.0000-0.3204	0.5063-0.9623
Maximum possible	0.1391	0.7343
Proportion of maximum possible	1	1

## Discussion

### Principal Findings

This study explored the feasibility of using NLP and machine learning to automate triage of patients with musculoskeletal conditions by analyzing information from referral letters. Specifically, we determined that LDA can automatically assort referral letters into topics that are clinically relevant. In other words, latent topics provide information that is considered relevant when prescribing treatments.

First, our experiments confirmed that latent topics could be used to automatically predict an appropriate treatment. A supervised classifier based on latent topics as its sole feature consistently outperformed the baseline method. Further improvements in the performance of such classifiers stand to be gained by incorporating other types of features that can be obtained from the patients' electronic health records, for example, demographics, body mass index, and imaging reports. However, this was beyond the scope of this study, which was concerned only with establishing the clinical relevance of automatically extracted latent topics. On their own, these topics proved to be sufficiently discriminative features for treatment recommendations based on machine learning.

Second, our experiments confirmed that latent topics could be interpreted by clinicians as cohorts of patients who share common characteristics or experiences such as medical history, demographics, and possible treatments. Specifically, the words associated with each topic by the LDA algorithm proved to be sufficiently descriptive to enable clinical specialists to interpret the topic's underlying semantics.

The first set of experiments established the clinical relevance of latent topics from a machine perspective: a treatment can be recommended automatically for an individual patient. The second set of experiments established the clinical relevance of latent topics from a human perspective: a treatment can be recommended by a clinician for an automatically identified cohort of patients. Both treatment recommendation scenarios support the hypothesis that topic modeling can support patient triage. Automating this process can be used to address areas where bottlenecks exist. Efficient referral to appropriate services such as analgesia or diagnostics not only improves patient experience and health outcomes but also reduces queuing arising from nonurgent demand, thus minimizing the delays for those with urgent care needs.

### Conclusions

Our approach used information contained in referral letters to underpin the referral decision-making process. Successful automation of this process has the potential to streamline care pathways and ensure that patients receive timely and optimal care. In clinical applications such as patient triage, interpretability is the key to build trust for all stakeholders, clinicians, and patients alike. Our approach to qualitative evaluation sets a precedent in measuring the interpretability of automated outputs, which is emerging as the next big challenge for clinical NLP. The unsupervised aspect of the proposed approach avoids the need for data annotation and, therefore, can be readily deployed to tackle other bottlenecks along the musculoskeletal pathway. For example, imaging and pathology reports can be processed in the same way to automatically redirect patients to the most appropriate services.

## Abbreviations

BOW: bag of words
CUI: concept unique identifier
LCP: log-conditional probability
LDA: latent Dirichlet allocation
NLP: natural language processing
NPMI: normalized pointwise mutual information
PMI: pointwise mutual information
TN: true negatives
TP: true positives
UMLS: Unified Medical Language System

## References

[1] Musculoskeletal Conditions. National Institute for Health and Care Excellence.

[2] Vos T, Flaxman AD, Naghavi M, Lozano R, Michaud C, Ezzati M, Shibuya K, Salomon JA, Abdalla S, Aboyans V, Abraham J, Ackerman I, Aggarwal R, Ahn SY, Ali MK, Alvarado M, Anderson HR, Anderson LM, Andrews KG, Atkinson C, Baddour LM, Bahalim AN, Barker-Collo S, Barrero LH, Bartels DH, Basáñez MG, Baxter A, Bell ML, Benjamin EJ, Bennett D, Bernabé E, Bhalla K, Bhandari B, Bikbov B, Bin Abdulhak A, Birbeck G, Black JA, Blencowe H, Blore JD, Blyth F, Bolliger I, Bonaventure A, Boufous S, Bourne R, Boussinesq M, Braithwaite T, Brayne C, Bridgett L, Brooker S, Brooks P, Brugha TS, Bryan-Hancock C, Bucello C, Buchbinder R, Buckle G, Budke CM, Burch M, Burney P, Burstein R, Calabria B, Campbell B, Canter CE, Carabin H, Carapetis J, Carmona L, Cella C, Charlson F, Chen H, Cheng AT, Chou D, Chugh SS, Coffeng LE, Colan SD, Colquhoun S, Colson KE, Condon J, Connor MD, Cooper LT, Corriere M, Cortinovis M, de Vaccaro KC, Couser W, Cowie BC, Criqui MH, Cross M, Dabhadkar KC, Dahiya M, Dahodwala N, Damsere-Derry J, Danaei G, Davis A, de Leo D, Degenhardt L, Dellavalle R, Delossantos A, Denenberg J, Derrett S, Des Jarlais DC, Dharmaratne SD, Dherani M, Diaz-Torne C, Dolk H, Dorsey ER, Driscoll T, Duber H, Ebel B, Edmond K, Elbaz A, Ali SE, Erskine H, Erwin PJ, Espindola P, Ewoigbokhan SE, Farzadfar F, Feigin V, Felson DT, Ferrari A, Ferri CP, Fèvre EM, Finucane MM, Flaxman S, Flood L, Foreman K, Forouzanfar MH, Fowkes FG, Franklin R, Fransen M, Freeman MK, Gabbe BJ, Gabriel SE, Gakidou E, Ganatra HA, Garcia B, Gaspari F, Gillum RF, Gmel G, Gosselin R, Grainger R, Groeger J, Guillemin F, Gunnell D, Gupta R, Haagsma J, Hagan H, Halasa YA, Hall W, Haring D, Haro JM, Harrison JE, Havmoeller R, Hay RJ, Higashi H, Hill C, Hoen B, Hoffman H, Hotez PJ, Hoy D, Huang JJ, Ibeanusi SE, Jacobsen KH, James SL, Jarvis D, Jasrasaria R, Jayaraman S, Johns N, Jonas JB, Karthikeyan G, Kassebaum N, Kawakami N, Keren A, Khoo JP, King CH, Knowlton LM, Kobusingye O, Koranteng A, Krishnamurthi R, Lalloo R, Laslett LL, Lathlean T, Leasher JL, Lee YY, Leigh J, Lim SS, Limb E (2012). Years lived with disability (YLDs) for 1160 sequelae of 289 diseases and injuries 1990-2010: a systematic analysis for the Global Burden of Disease Study 2010. Lancet.

[3] Johnson SA, Kalairajah Y, Moonot P, Steele N, Field RE (2008). Fast-track assessment clinic: selection of patients for a one-stop hip assessment clinic. Ann R Coll Surg Engl.

[4] Inglis T, Armour P, Inglis G, Hooper G (2017). Rationing of hip and knee referrals in the public hospital: the true unmet need. N Z Med J.

[5] Spasić I, Zhao B, Jones C, Button K (2015). KneeTex: an ontology-driven system for information extraction from MRI reports. J Biomed Semantics.

[6] Hassanpour S, Langlotz C, Amrhein T, Befera N, Lungren M (2017). Performance of a machine learning classifier of knee MRI reports in two large academic radiology practices: a tool to estimate diagnostic yield. AJR Am J Roentgenol.

[7] Spasić Irena, Owen D, Smith A, Button K (2019). KLOSURE: closing in on open-ended patient questionnaires with text mining. J Biomed Semantics.

[8] Spasić I, Uzuner O, Zhou L (2020). Emerging clinical applications of text analytics. Int J Med Inform.

[9] Spasić I, Livsey J, Keane JA, Nenadić G (2014). Text mining of cancer-related information: review of current status and future directions. Int J Med Inform.

[10] Spasic I, Nenadic G (2020). Clinical text data in machine learning: systematic reviews. JMIR Med Inform.

[11] Holzinger A, Langs G, Denk H, Zatloukal K, Müller H (2019). Causability and explainability of artificial intelligence in medicine. Wiley Interdiscip Rev Data Min Knowl Discov.

[12] Blei D, Ng A, Jordan M (2003). Latent Dirichlet Allocation. J Mach Learn Res.

[13] Huang Z, Lu X, Duan H (2013). Latent treatment pattern discovery for clinical processes. J Med Syst.

[14] Arnold CW, Oh A, Chen S, Speier W (2016). Evaluating topic model interpretability from a primary care physician perspective. Comput Methods Programs Biomed.

[15] Wang L, Wang Y, Shen F, Rastegar-Mojarad M, Liu H (2019). Discovering associations between problem list and practice setting. BMC Med Inform Decis Mak.

[16] Rumshisky A, Ghassemi M, Naumann T, Szolovits P, Castro VM, McCoy TH, Perlis RH (2016). Predicting early psychiatric readmission with natural language processing of narrative discharge summaries. Transl Psychiatry.

[17] Fong A, Ratwani R (2015). An evaluation of patient safety event report categories using unsupervised topic modeling. Methods Inf Med.

[18] Bisgin H, Liu Z, Fang H, Xu X, Tong W (2011). Mining FDA drug labels using an unsupervised learning technique--topic modeling. BMC Bioinformatics.

[19] Sullivan R, Sarker A, O'Connor K, Goodin A, Karlsrud M, Gonzalez G (2016). Finding Potentially Unsafe Nutritional Supplements From User Reviews With Topic Modeling. Pacific Symposium on Biocomputing.

[20] Chen Y, Ghosh J, Bejan CA, Gunter CA, Gupta S, Kho A, Liebovitz D, Sun J, Denny J, Malin B (2015). Building bridges across electronic health record systems through inferred phenotypic topics. J Biomed Inform.

[21] Zech J, Pain M, Titano J, Badgeley M, Schefflein J, Su A, Costa A, Bederson J, Lehar J, Oermann EK (2018). Natural language-based machine learning models for the annotation of clinical radiology reports. Radiology.

[22] Barroilhet SA, Pellegrini AM, McCoy TH, Perlis RH (2020). Characterizing DSM-5 and ICD-11 personality disorder features in psychiatric inpatients at scale using electronic health records. Psychol Med.

[23] Button K, Spasić I, Playle R, Owen D, Lau M, Hannaway L, Jones S (2020). Using routine referral data for patients with knee and hip pain to improve access to specialist care. BMC Musculoskelet Disord.

[24] Spasic I, Krzeminski D, Corcoran P, Balinsky A (2019). Cohort selection for clinical trials from longitudinal patient records: text mining approach. JMIR Med Inform.

[25] Griffis D, Shivade C, Fosler-Lussier E, Lai A (2016). A quantitative and qualitative evaluation of sentence boundary detection for the clinical domain. AMIA Jt Summits Transl Sci Proc.

[26] Pakhomov S, Pedersen T, Chute C (2005). Abbreviation and acronym disambiguation in clinical discourse. AMIA Annu Symp Proc.

[27] Spasić Irena, Greenwood M, Preece A, Francis N, Elwyn G (2013). FlexiTerm: a flexible term recognition method. J Biomed Semantics.

[28] Spasic I (2018). Acronyms as an integral part of multi-word term recognition – a token of appreciation. IEEE Access.

[29] Documentation. NLTK Project.

[30] Named Entity Recognition. Explosion AI.

[31] Button K, van Deursen RW, Soldatova L, Spasić I (2013). TRAK ontology: defining standard care for the rehabilitation of knee conditions. J Biomed Inform.

[32] Bodenreider O (2004). The unified medical language system (UMLS): integrating biomedical terminology. Nucleic Acids Res.

[33] Wang X, McCallum A, Wei X (2007). Topical N-grams: Phrase and Topic Discovery, With an Application to Information Retrieval. Seventh IEEE International Conference on Data Mining.

[34] Aronson AR, Lang F (2010). An overview of MetaMap: historical perspective and recent advances. J Am Med Inform Assoc.

[35] (2020). Semantic Type Mappings. MetaMap Documentation.

[36] Rehurek R, Sojka P (2010). Software Framework for Topic Modelling with Large Corpora. LREC Workshop on New Challenges for NLP Frameworks.

[37] Asuncion A, Welling M, Smyth P, Teh Y (2009). On Smoothing and Inference for Topic Models. 25th Conference on Uncertainty in Artificial Intelligence.

[38] Wallach H, Murray I, Salakhutdinov R, Mimno D (2009). Evaluation Methods for Topic Models. 26th Annual International Conference on Machine Learning.

[39] Zhao W, Chen JJ, Perkins R, Liu Z, Ge W, Ding Y, Zou W (2015). A heuristic approach to determine an appropriate number of topics in topic modeling. BMC Bioinformatics.

[40] Chang J, Boyd-Graber J, Gerrish S, Wang C, Blei D (2009). Reading Tea Leaves: How Humans Interpret Topic Models. 22nd International Conference on Neural Information Processing Systems.

[41] Roder M, Both A, Hinneburg A (2015). Exploring the Space of Topic Coherence Measures. 8th ACM International Conference on Web Search and Data Mining.

[42] Newman D, Lau JH, Grieser K, Baldwin T (2010). Automatic Evaluation of Topic Coherence. Conference of the North American Chapter of the Association for Computational Linguistics.

[43] Church K, Hanks P (1989). Word Association Norms, Mutual Information, and Lexicography. 27th Annual Meeting of the Association for Computational Linguistics.

[44] Bouma G (2009). Normalized (Pointwise) Mutual Information in Collocation Extraction. Conference of the German Society for Computational Linguistics and Language Technology.

[45] Michelbacher L, Evert S, Schütze H (2007). Asymmetric Association Measures. International Conference on Recent Advances in Natural Language Processing.

[46] Aletras N, Stevenson M (2013). Evaluating Topic Coherence Using Distributional Semantics. 10th International Conference on Computational Semantics.

[47] Mimno D, Wallach H, Talley E, Leenders M, McCallum A (2011). Optimizing Semantic Coherence in Topic Models. Conference on Empirical Methods in Natural Language Processing.

[48] Sievert C, Shirley K (2014). A Method for Visualizing and Interpreting Topics. Workshop on Interactive Language Learning, Visualization and Interfaces.

[49] Cohen J (2016). A coefficient of agreement for nominal scales. Educ Psychol Measure.

[50] Cohen J (1968). Weighted kappa: nominal scale agreement with provision for scaled disagreement or partial credit. Psychol Bull.

[51] Fleiss JL, Cohen J, Everitt BS (1969). Large sample standard errors of kappa and weighted kappa. Psychol Bull.

[52] Fleiss J, Cohen J (1973). The equivalence of weighted kappa and the intraclass correlation coefficient as measures of reliability. Educ Psychol Measure.

